# A Phase 3 Randomized Trial Investigating the Safety, Tolerability, and Immunogenicity of V116, an Adult-Specific Pneumococcal Vaccine, Compared with PPSV23, in Adults ≥50 Years of Age (STRIDE-10)

**DOI:** 10.3390/vaccines13040341

**Published:** 2025-03-22

**Authors:** Veronika Jotterand, Vinita Jagannath, Andrea Accini Diaz, Juan Diego Velez, Arna Letica, Silvia Narejos Perez, Rebecca Clark, Yoseph Caraco, Olaf Degen, Kyung-Hwa Park, Serhat Ünal, Frederick Wittke, Kimberly Hurtado, Clay Churchill, Ying Zhang, Doreen Fernsler, Jianing Li, Ulrike K. Buchwald, Heather Platt

**Affiliations:** 1 MSD, 6005 Lucerne, Switzerland; veronika.jotterand@msd.com; 2 MSD (UK) Limited, 120 Moorgate, London EC2M 6UR, UK; 3 IPS Centro Cientifico Asistencial S.A.S, Barranquilla 080020, Colombia; andreacciniipscca@gmail.com; 4 Fundacion Valle del Lili, Cali 760032, Colombia; juan.velez.lo@fvl.org.co; 5 Optimal Clinical Trials, Auckland 1010, New Zealand; arna@optimalclinicaltrials.com; 6 CAP Centelles, 08540 Barcelona, Spain; snarejos@ebacentelles.cat; 7 Layton Medical Centre, Flyde Coast Clinical Research, 200 Kingscote Drive, Blackpool FY3 7EN, UK; rebecca.clark26@nhs.net; 8 Division of Medicine, Hadassah Medical Center, Faculty of Medicine, Hebrew University, Jerusalem 91120, Israel; caraco.yoseph@mail.huji.ac.il; 9 University Medical Center Hamburg-Eppendorf, Martinistraße 52, 20246 Hamburg, Germany; degen@uke.de; 10 Chonnam National University Medical School, Department of Infectious Diseases, Gwangju 61469, Republic of Korea; iammedkid@naver.com; 11 Department of Infectious Diseases and Clinical Microbiology, Hacettepe University School of Medicine, 06100 Ankara, Turkey; sunal@hacettepe.edu.tr; 12 MSD, The Circle 66, Zürich Airport, 8058 Zürich, Switzerland; frederick.wittke@msd.com; 13 Merck & Co., Inc., Rahway, NJ 07065, USA; kimberly_tucker2@merck.com (K.H.); clay.churchill@merck.com (C.C.); ying.zhang30@merck.com (Y.Z.); doreen_fernsler@merck.com (D.F.); jianing.li@merck.com (J.L.); ulrike.buchwald@merck.com (U.K.B.); heather.platt@merck.com (H.P.)

**Keywords:** pneumococcal conjugate vaccine, serotype, adult, opsonophagocytic activity, invasive pneumococcal disease

## Abstract

**Background**: V116 is a 21-valent pneumococcal conjugate vaccine (PCV) designed for adults. It contains the most prevalent serotypes associated with invasive pneumococcal disease (IPD) in adults in regions with established pediatric vaccination programs. This Phase 3 study compared the safety, tolerability, and immunogenicity of V116 with 23-valent pneumococcal polysaccharide vaccine (PPSV23) in adults ≥50 years of age. **Methods**: In this randomized, active comparator-controlled, parallel-group, multisite, double-blind study, participants were randomized 1:1 to receive a single dose of V116 or PPSV23 (NCT05569954). Primary immunogenicity outcomes assessed the opsonophagocytic activity (OPA) responses for (i) non-inferiority for 12 serotypes common to V116 and PPSV23 based on V116/PPSV23 geometric mean titers (GMTs) at Day 30, and (ii) superiority for nine serotypes unique to V116 using V116/PPSV23 GMTs and the proportions of participants with a ≥4-fold rise in OPA responses from baseline to 30 days post-vaccination. The primary safety outcome was evaluated as the proportion of participants with solicited injection-site and systemic adverse events through Day 5 post-vaccination, and vaccine-related serious adverse events up to 6 months post-vaccination. **Findings**: V116 was non-inferior to PPSV23 for all 12 common serotypes, superior to PPSV23 for all nine unique serotypes based on V116/PPSV23 GMTs, and superior to PPSV23 for eight of the nine serotypes unique to V116, based on the proportion of participants with a ≥4-fold rise in OPA responses (except for serotype 15C). The safety profile of V116 was comparable to that of PPSV23. **Interpretation**: In regions with established vaccination programs, V116 could broaden the serotype coverage for residual pneumococcal disease in adults.

## 1. Introduction

In adults, bacterial community-acquired pneumonia (CAP) is most commonly caused by *Streptococcus pneumoniae* and is associated with substantial morbidity, mortality, and economic burden [1,2,3,4,5].

Between 2016 and 2019, among US adults 18–49, 50–64, and ≥65 years of age, the rates of all-cause pneumonia per 100,000 patient-years were 953, 2679, and 6930, respectively [6]. Studies have found that *S. pneumoniae* causes 10% to 37% of CAP requiring hospitalization in adults in Europe and the United States [5,7,8,9]. In the United States, pneumococcal pneumonia causes approximately 100,000 hospitalizations among adults per year [10]. Invasive pneumococcal disease (IPD), defined by the isolation of *S. pneumoniae* in otherwise sterile sites, is associated with high mortality among adults ≥50 years of age, with the highest mortality rate observed in adults ≥75 years of age [11]. Available data from the US Centers for Disease Control and Prevention (CDC) estimated that, while the rates of IPD decreased during the coronavirus disease 2019 (COVID-19) pandemic, the frequency of the disease is now returning to pre-pandemic levels in adults ≥19 years of age [12,13].

Pneumococcal conjugate vaccines (PCVs) with varying serotype coverage, as well as the 23-valent pneumococcal polysaccharide vaccine (PPSV23; Pneumovax^®^23, Merck Sharp & Dohme LLC, a subsidiary of Merck & Co., Inc., Rahway, NJ, USA [MSD]), are approved for use in adults in many countries [14]. These vaccines are immunogenic and effective, with a favorable safety profile, and can be used to decrease respiratory disease burden, cases of IPD, and healthcare utilization [15,16,17]. An increasing incidence of pneumococcal disease (PD) due to serotypes not historically or currently included in licensed PCVs has been observed, including in adults [18,19,20].

V116 (CAPVAXIVE; approved by the US Food and Drug Administration on 17 June 2024) is a 21-valent PCV specifically designed for use in adults [21]. In countries with routine PCV vaccination of infants and children, the 21 serotypes included in V116 are the most prevalent causes of IPD in adults [12,18,19,22,23]. Given the indirect protection provided through infant vaccination programs, as well as the variations in serotype distribution between adults and children, a vaccine specifically targeting the serotypes linked to residual adult disease could be a more effective preventive strategy for adults [24]. V116 is designed to complement pediatric immunization programs that provide indirect protection in adults.

Serotypes in V116 are responsible for ~83% and ~84% of residual IPD in adults 50–64 and ≥65 years of age, respectively (pre-COVID-19 pandemic data) [12,13]. V116 contains eight unique serotypes not included in other currently licensed PCVs, which accounted for ~30% of IPD in US adults ≥65 years of age in 2019 [1,25]. Among adults ≥18 years of age enrolled in a Phase 1/2 trial, V116 had a safety profile that was generally comparable to that of PPSV23. The trial demonstrated non-inferiority of V116 for serotypes common to both vaccines, and superiority for the serotypes in V116 but not PPSV23 [25]. PPSV23 was chosen as an active comparator due to it having the highest number of shared serotypes with V116 and because it is among the most widely used pneumococcal vaccines in adults globally, with 40 years of clinical experience [26,27,28]. In a Phase 3 trial of adults ≥50 years of age who were pneumococcal-vaccine experienced, V116 demonstrated a similar safety profile to PPSV23 and 15-valent PCV (PCV15), and was immunogenic [29].

This Phase 3 clinical study was conducted to evaluate the safety, tolerability, and immunogenicity of V116 in adults ≥50 years of age who had not previously received a pneumococcal vaccine. Older adults are at an increased risk of PD and associated morbidity and mortality due to aging-related physiological changes in the respiratory system, age-related immunosenescence, and an increased incidence of other medical conditions associated with an increased risk of IPD [6,30,31].

## 2. Methods

### 2.1. Study Design

This study (Protocol #V116-010; registered at ClinicalTrials.gov as NCT05569954, on 4 October 2022) [32] was a randomized, active comparator-controlled, parallel-group, multisite, double-blind study to evaluate the safety, tolerability, and immunogenicity of V116 in pneumococcal vaccine-naïve adults ≥50 years of age. The study was conducted across 55 study sites in 11 countries (Argentina, Australia, Colombia, Germany, Israel, New Zealand, South Korea, Spain, Taiwan, Turkey, and the United Kingdom).

The study was conducted in accordance with principles of Good Clinical Practice and was approved by the appropriate institutional review boards and regulatory agencies [33].

The study protocol and statistical analysis plan are available online (https://cdn.clinicaltrials.gov/large-docs/54/NCT05569954/Prot_SAP_000.pdf; accessed on 16 March 2025) [32].

### 2.2. Participants, Randomization, and Blinding

Eligible participants were adults ≥50 years of age who had not previously received any pneumococcal vaccine. The aim of recruitment was to ensure that at least 50% of participants were ≥65 years of age. Participants with underlying chronic conditions had to be in a stable condition, according to investigator judgment. Participants were excluded if they had a history of IPD, known or suspected impairment of immunological function, or had received systemic corticosteroids (prednisone equivalent of ≥20 mg/day for 14 consecutive days and had not completed the intervention at least 14 days prior to receiving the study vaccine) or any form of current immunosuppressive therapy.

**Figure 1:**
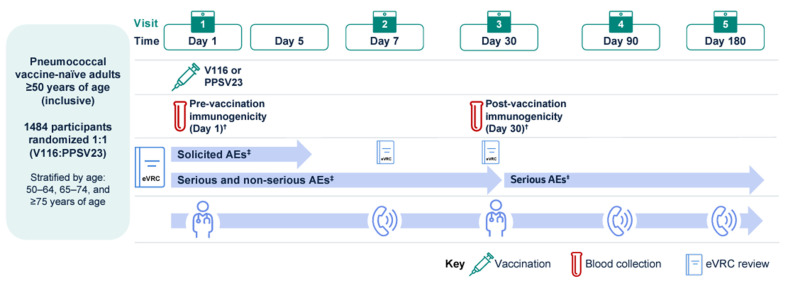
Study design. ^†^ The per-protocol population included all randomized participants without protocol deviations that could have substantially affected the results of the immunogenicity analyses. ^‡^ The safety population included all randomized participants who received study intervention. AE: adverse event; eVRC: electronic vaccination report card; PPSV23: 23-valent pneumococcal polysaccharide vaccine; V116: 21-valent, adult-specific pneumococcal conjugate vaccine.

Participants were randomly assigned 1:1 to receive a single intramuscular dose of either V116 or PPSV23 (Figure 1). Randomization and its implementation occurred centrally using an interactive response technology system, and was stratified by age at enrollment (50–64 years, 65–74 years, and ≥75 years). The study sponsor generated the randomized allocation schedules. Participants, investigators, and sponsor personnel were blinded to the intervention. As V116 and PPSV23 have a different appearance, an unblinded pharmacist (or other qualified study-site personnel) was responsible for receiving, maintaining, preparing and/or dispensing, and administering study vaccines. To avoid bias, contact between the unblinded study site personnel and study participants was otherwise prohibited, and blinded personnel were responsible for all other study procedures and assessments.

### 2.3. Vaccines

V116 (Capvaxive™, MSD) is a licensed 21-valent PCV approved in the United States and Canada, indicated in individuals ≥18 years of age for active immunization to prevent invasive disease and pneumonia caused by the following serotypes of *S. pneumoniae*: 3, 6A, 7F, 8, 9N, 10A, 11A, 12F, 15A, 15C, 16F, 17F, 19A, 20A, 22F, 23A, 23B, 24F, 31, 33F, and 35B, and also for the prevention of invasive disease caused by serotype 15B. These serotypes are all individually conjugated to the carrier protein CRM197. The de-O-acetylated 15B (deOAc15B) capsular pneumococcal polysaccharide (PnPs) is used to elicit antibodies to serotype 15C, as it is structurally similar to 15C PnPs [34,35,36].

PPSV23 (Pneumovax^®^23, MSD) is a licensed 23-valent pneumococcal polysaccharide vaccine recommended for adults ≥65 years of age and individuals ≥2 years of age with certain medical conditions for the prevention of PD. PPSV23 contains 23 different serotypes: 1, 2, 3, 4, 5, 6B, 7F, 8, 9N, 9V, 10A, 11A, 12F, 14, 15B, 17F, 18C, 19A, 19F, 20, 22F, 23F, and 33F [15,37,38,39].

V116 and PPSV23 contain 12 common serotypes. Advances in serotyping methods led to further characterization of serotype 20 included in PPSV23 as serotype 20A [40]; this serotype is represented as serotype 20 in PPSV23 labeling. The V116 formulation includes 20A; immune responses to serotype 20A are assessed in this study.

### 2.4. Immunogenicity Assessments

Blood samples were taken prior to vaccination (Day 1) and 30 days post-vaccination (Day 30) to measure immune responses. Assays were developed at MSD and validated for use in the V116 clinical trial program. A microcolony multiplexed opsonophagocytic killing assay (mMOPA) [41] was used to measure functional antibodies, and an electrochemiluminescence (ECL) assay using custom plates from Meso Scale Discovery (Rockville, MD, USA) was used to measure serotype-specific PnPs immunoglobulin G (IgG) antibodies [42].

### 2.5. Safety Assessments

Participants used an electronic vaccination report card (eVRC) to record adverse events (AEs) following vaccination. The investigator subsequently assessed and reported all recorded AEs. Solicited AEs, including both injection-site and systemic AEs, were recorded from Day 1 to Day 5 post-vaccination. Unsolicited AEs were collected for 30 days post-vaccination and serious AEs (SAEs) for the duration of study participation. On-site visits occurred on Day 1 and Day 30, while telephone reviews were conducted on Day 7, Day 90, and Day 180 (Figure 1).

### 2.6. Outcomes

The primary immunogenicity outcomes were to assess opsonophagocytic activity (OPA) responses to V116 for non-inferiority or superiority to PPSV23. Non-inferiority was assessed for serotypes common to V116 and PPSV23, and superiority for serotypes unique to V116. Non-inferiority was based on V116/PPSV23 geometric mean titers (GMTs) at Day 30, and superiority was based on GMTs at Day 30 and the proportions of participants with a ≥4-fold rise in OPA responses from baseline to 30 days post-vaccination.

Secondary immunogenicity outcomes were to evaluate the serotype-specific IgG geometric mean concentrations (GMCs) at 30 days post-vaccination with V116 and PPSV23, the proportions of participants with a ≥4-fold rise in serotype-specific cross-reactive OPA responses from baseline to 30 days post-vaccination for serotypes within a serogroup in V116, and the serotype-specific geometric mean fold rise (GMFR) and proportions of participants with a ≥4-fold rise in serotype-specific OPA and IgG responses from baseline to 30 days post-vaccination for V116 and separately for PPSV23.

The primary safety outcomes were the proportions of participants with solicited AEs from Day 1 to Day 5, including both injection-site and solicited systemic AEs, and the proportions of participants with vaccine-related SAEs from Day 1 through ~6 months following vaccination.

### 2.7. Statistical Analyses

Approximately 1400 participants were planned to be enrolled in this study; 700 participants were randomized into each of the V116 and PPSV23 groups, to provide 90% power for all primary immunogenicity objectives. The assumed true underlying OPA GMT ratios for common and unique serotypes and the proportions of participants with a ≥4-fold rise in OPA responses from baseline to 30 days post-vaccination were based on prior studies, with the power for the primary hypotheses calculated using a simulation-based approach. The immunogenicity analyses were based on the per-protocol population. This included participants who were randomized and did not have any protocol deviations that could have substantially affected the results of the immunogenicity endpoints.

The statistical criterion for non-inferiority required the lower bound of the two-sided 95% confidence interval (CI) of the OPA GMT ratio V116/PPSV23, calculated using the constrained longitudinal data analysis (cLDA) method, to be >0.5 [43]. The statistical criterion for superiority required the lower bound of the two-sided 95% CI of the OPA GMT ratio V116/PPSV23, calculated using the cLDA method, to be >2.0 [43]. A cLDA method proposed by Liang and Zeger [44] was used to compare serotype-specific OPA GMTs at 30 days post-vaccination between the two vaccination groups. In this model, the response vector consisted of the log-transformed antibody titers at baseline and 30 days post-vaccination. The repeated measures model includes terms for time, vaccination group, the interaction of time-by-vaccination group, age stratum (i.e., 50–64 years, 65–74 years, and ≥75 years of age) at vaccination, and the interaction of time-by-age stratum. This model allows for different baseline means for each age stratum, but restricts the baseline mean within each age stratum to be the same for all vaccination groups.

Superiority was also assessed as the lower bound of the two-sided 95% CI of the differences (V116−PPSV23) between the proportions of participants with a ≥4-fold rise in serotype-specific OPA responses, calculated using the stratified Miettinen–Nurminen (MN) method [45], to be >10%. The stratified MN method is an unconditional, asymptotic method [45] that estimates the difference in proportions for each stratum (e.g., age groups: 50–64 years, 65–74 years, and ≥75 years at vaccination) between the two vaccination groups and provides the overall difference along with its two-sided 95% CI, weighted by sample size.

The acceptability criteria for a cross-reactive antibody response to serotypes 6C and 15B required the lower bound of the 95% CI of the proportions of participants with a ≥4-fold rise in serotype-specific OPA responses from baseline to Day 30 for V116, computed using the exact binomial method of Clopper and Pearson, to be >0.5 [46].

Subgroup analyses were conducted for OPA GMT ratios at Day 30 using the cLDA method and the proportions of participants with a ≥4-fold rise in serotype-specific OPA responses using the stratified MN method [43,45].

The statistical testing criteria for non-inferiority and superiority were selected based on regulatory precedent for comparing post-vaccination antibody levels between licensed and investigational pneumococcal vaccines [23,47,48].

Safety and tolerability were assessed by clinical review of all relevant parameters, including AEs and post-vaccination temperature measurements. Descriptive statistics were provided by intervention groups. For select safety parameters, between-group 95% CIs were provided for the percentage of participants using the MN method. Baseline characteristics were summarized via descriptive statistics.

## 3. Results

### 3.1. Study Population

**Figure 2:**
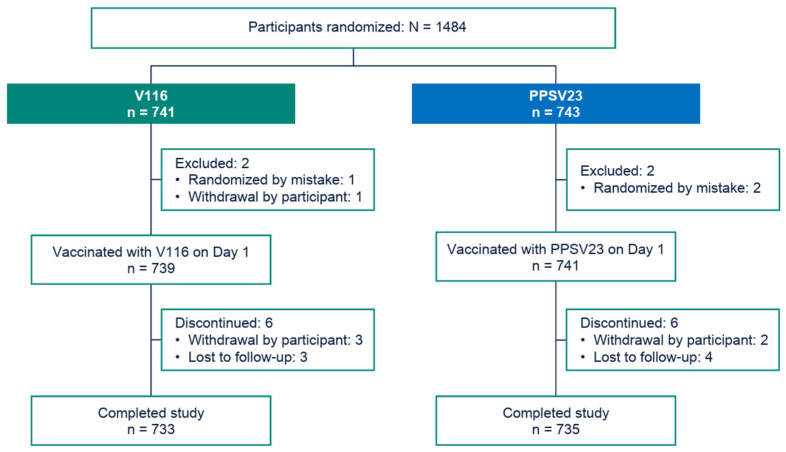
CONSORT flow diagram. Each participant was counted once for trial disposition based on the latest corresponding disposition record. The reasons for screened participants not undergoing randomization were screen failures (n = 43), withdrawal by participants (n = 2), and other (n = 1). PPSV23: 23-valent pneumococcal polysaccharide vaccine; V116: 21-valent, adult-specific pneumococcal conjugate vaccine.

The study was conducted from November 2022 to October 2023. The disposition of participants was generally comparable between the V116 and PPSV23 intervention groups (Figure 2). A total of 1484 participants were randomized, and 1480 participants were vaccinated. The majority of participants completed the study (>98%). The most common reason for discontinuing study participation across both intervention groups was loss to follow-up (n = 7; three in the V116 group, and four in the PPSV23 group) (Figure 2).

### 3.2. Baseline Characteristics and Demographics

In general, the demographic characteristics of the study population were comparable between the V116 and PPSV23 groups (Table 1). The majority of the participants were female, White, and not of Hispanic or Latino ethnicity (Table 1). The overall median age was 65 years, with most participants (54%) being ≥65 years of age (Table 1). A similar proportion of participants in both the V116 and PPSV23 groups had 0, 1, or ≥2 risk factors associated with an increased risk of PD. In addition, the proportions of participants with specific risk factors were generally comparable across groups (Supplementary Table S1).

### 3.3. Immunogenicity

**Figure 3:**
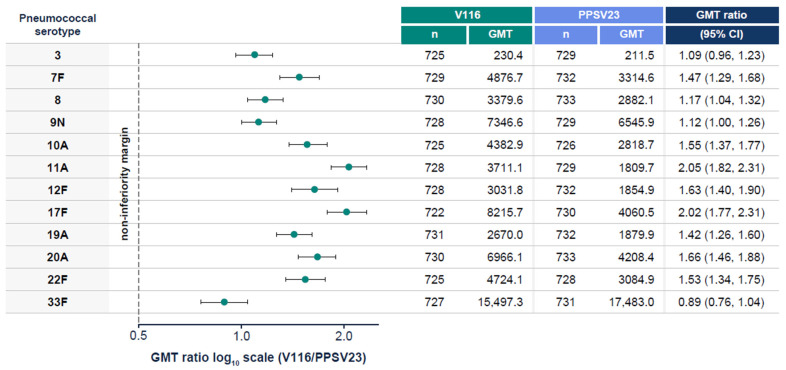
OPA GMTs for all 12 common serotypes at 30 days post-vaccination. Non-inferiority = lower bound of the 95% CI for the estimated GMT ratio (V116/PPSV23) > 0.5 (1-sided *p*-value < 0.025). CI: confidence interval; GMT: geometric mean titer; OPA: opsonophagocytic activity; PPSV23: 23-valent pneumococcal polysaccharide vaccine; V116: 21-valent, adult-specific pneumococcal conjugate vaccine.

**Figure 4:**
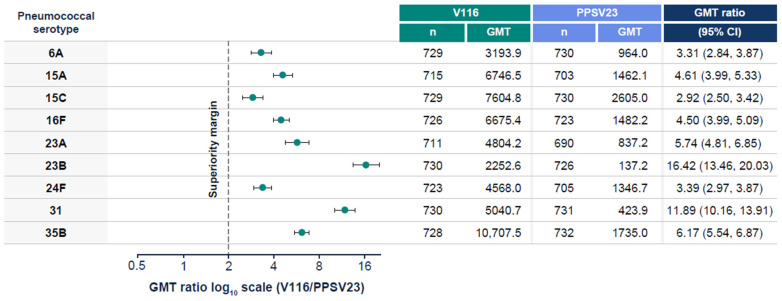
OPA GMTs for all nine unique serotypes at 30 days post-vaccination. Superiority = lower bound of the 95% CI for estimated GMT ratio (V116/PPSV23) > 2.0 (1-sided *p*-value < 0.025). CI: confidence interval; GMT: geometric mean titer; OPA: opsonophagocytic activity; PPSV23: 23-valent pneumococcal polysaccharide vaccine; V116: 21-valent, adult-specific pneumococcal conjugate vaccine.

**Figure 5:**
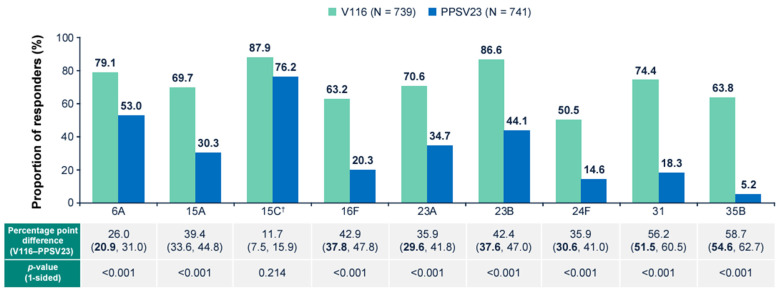
Percentage of participants with a 4-fold rise in OPA Day 1 to Day 30 for all nine unique serotypes. Superiority = lower bound of the 95% CI for the difference in response rates (V116–PPSV23) ≥ 10% (1-sided *p*-value < 0.025). Estimated difference, 95% CI, and *p*-value are based on the stratified Miettinen & Nurminen method. ^†^ Serotype 15C represents the immune response to the deOAc15B polysaccharide, as the molecular structure for deOAc15B and 15C are similar; anti-15C immune responses are assessed in this study. CI: confidence interval; OPA: opsonophagocytic activity; PPSV23: 23-valent pneumococcal polysaccharide vaccine; V116: 21-valent, adult-specific pneumococcal conjugate vaccine.

V116 was non-inferior to PPSV23 for all 12 common serotypes, based on OPA GMT analysis at 30 days post-vaccination (Figure 3). V116 was superior to PPSV23 for all nine unique serotypes, based on OPA GMT analysis at 30 days post-vaccination (Figure 4). V116 was superior to PPSV23 for eight of nine serotypes unique to V116 (except for serotype 15C), as assessed by the proportion of participants with a ≥4-fold rise in OPA from baseline to 30 days post-vaccination (Figure 5).

V116 met the pre-defined criterion for an acceptable cross-reactive antibody response for serotype 15B, based on the proportion of participants with a 4-fold rise in OPA from Day 1 to Day 30. Serotype 6C did not meet the criterion for an acceptable antibody response; the lower bound of the 95% CI was 48.7% (Supplementary Table S2).

Between-group comparisons of IgG GMCs at 30 days post-vaccination for both common and unique serotypes were consistent with the results of the primary analysis of OPA GMTs (Supplementary Figures S1 and S2). The observed serotype-specific GMFRs at 30 days post-vaccination were generally comparable in both intervention groups for the 12 serotypes common to V116 and PPSV23 and were higher for the nine serotypes unique to V116 (Supplementary Figure S3).

For primary immunogenicity endpoints, serotype-specific OPA GMT ratios (Supplementary Figure S4) and the proportions of participants with a ≥4-fold rise in serotype-specific OPA responses for the nine unique serotypes to V116 at 30 days post-vaccination within the subgroup, stratified by age (50–64 years, 65–74 years, and ≥75 years), were generally consistent with the results observed in the overall population (Supplementary Figure S4).

### 3.4. Safety

**Figure 6:**
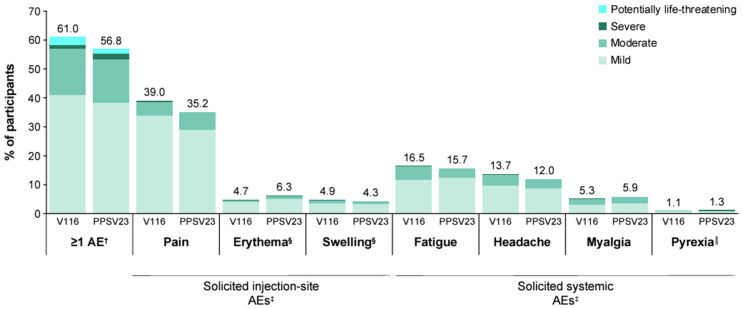
Summary of AEs by severity following vaccination. ^†^ Non-serious AEs were collected from Day 1 to Day 30 post-vaccination and SAEs were collected from Day 1 through the duration of study participation. ^‡^ Solicited injection-site AEs and solicited systemic AEs were collected from Day 1 to Day 5 post-vaccination. **^§^** Erythema and swelling were graded according to size and presented as intensity grade, as follows: mild (0 to ≤5.0 cm); moderate (>5.0 to ≤10.0 cm); and severe (>10.0 cm). ^║^ Pyrexia was defined as maximum temperature ≥100.4 °F (38.0 °C), with ≥104.0 °F (40.0 °C) defined as potentially life-threatening pyrexia. AE: adverse event; PPSV23: 23-valent pneumococcal polysaccharide vaccine; SAE: serious adverse event; V116: 21-valent, adult-specific pneumococcal conjugate vaccine.

The proportions of participants with AEs, including injection-site AEs, systemic AEs, vaccine-related AEs, and SAEs, were comparable between the V116 and PPSV23 intervention groups; 61.0% and 56.8% had ≥1 AEs in the V116 and PPSV23 groups, respectively (Table 2). The most frequently reported (≥5%) AEs were solicited AEs (injection-site pain, headache, fatigue, injection-site erythema, and myalgia) in both intervention groups; injection-site swelling was also reported in ≥5% of participants in the V116 intervention group. The V116 and PPSV23 groups had generally comparable proportions of participants with solicited AEs (49.9% vs. 48.4%) (Table 2). Of the participants with solicited AEs, the majority had events that were of mild-to-moderate intensity (Table 2; Figure 6) and of short duration (≤3 days) in both the V116 and PPSV23 groups (Supplementary Table S3). None of the solicited AEs were potentially life-threatening (Grade 4).

No vaccine-related SAEs were reported in the study. There were no deaths due to AEs reported in the study (Table 2).

A trend towards lower proportions of participants with AEs was observed in participants 65–74 and ≥75 years of age compared with participants 50–64 years of age for both interventions (Supplementary Table S4). Safety results for the subgroups stratified by age were generally consistent with the overall population (Supplementary Table S5).

## 4. Discussion

This study was conducted as part of the V116 clinical development program to evaluate an adult-specific PCV to address an unmet medical need in adults. PPSV23 was included as the comparator because it contains the largest number of serotypes in common with V116, and, globally, it remains a commonly used pneumococcal vaccine for adults [49,50,51].

This study showed that V116 was non-inferior to PPSV23 for all 12 of the serotypes common to both vaccines and was superior to PPSV23 for all nine unique serotypes in V116, based on the analysis of OPA GMT ratios at 30 days post-vaccination. V116 was also superior to PPSV23 for eight of the nine unique serotypes in V116, based on the proportions of participants who achieved a ≥4-fold rise in OPA responses from baseline to 30 days post-vaccination. OPA responses (including GMTs, GMFRs, and proportions of participants with a ≥4-fold rise) are considered an established immunologic surrogate endpoint, and, in the absence of a correlate of protection in adults, the totality of immune responses observed support that vaccination with V116 may result in protection against PD.

V116 did not meet the superiority criterion for serotype 15C, as assessed by the proportion of participants who achieved a ≥4-fold rise in OPA responses from baseline to 30 days post-vaccination. However, serotype 15C elicited the greatest ≥4-fold rise in OPA responses (87.9%) and OPA GMFR (49.4) across all unique serotypes in V116. The consistency of the immune response against serotype 15C across the endpoints provides confidence in the expected vaccine performance and that V116 will provide protection against serotype 15C [52]. In addition, as the immune response to serotype 15C in the PPSV23 group is likely attributed to cross-reactivity based on the presence of 15B antigen in PPSV23, evaluation of cross-reactivity is an important consideration in the comparative assessment of immune responses [53,54,55]. Cross-reactive immune responses to serotype 6C were observed in the V116 group, attributed to the presence of serotype 6A in the vaccine. While the pre-defined acceptable antibody response criterion for cross-reactive serotype 6C was not met, the totality of immune responses supports that V116 may provide cross-protection. Cross-protection against serotype 6C has been previously established following the introduction of 13-valent PCV (PCV13) [56]. This cross-protection is assumed by surveillance systems, including the CDC Active Bacterial Core surveillance (ABCs), for IPD incidence reporting [57].

The safety results from this study show that V116 is well tolerated in adults ≥50 years of age, with a safety profile that is generally comparable to PPSV23, which is consistent with the results observed in other PCV studies [47,58].

The proportions of participants with AEs were generally comparable across the V116 and PPSV23 intervention groups in the overall population and in the age subgroups analyzed. Lower proportions of participants with AEs were observed in the older age groups (65–74 and ≥75 years of age) compared with younger age groups (50–64 years of age) for both interventions, potentially due to less reactogenicity in older adults due to immune-senescence [30,31,59,60]. Solicited AEs, including both injection-site and solicited systemic AEs, were recorded from Day 1 to Day 5 post-vaccination. Based, in part, on the results from the Phase 1/2 study evaluating V116 in adults compared with PPSV23 [25], a 5-day solicited AE reporting period was assessed to be adequate in this study; this time frame was used across all studies in the V116 Phase 3 program. In addition, participants could report any AE through 30 days post-vaccination, and SAEs were reported throughout the duration of participation in the study.

This trial enrolled participants from 55 sites in 11 countries or territories. All trial sites participating in this study received training on study procedures. A dedicated central laboratory was used for testing blood samples for immunogenicity endpoints. Therefore, site-specific variability was minimized. In addition, including participants from various locations enhances generalizability and ensures that our results are representative of a wide range of populations.

PPSV23 was selected as a comparator based on several considerations, including its status as the standard of care, including in Europe, at the time of the STRIDE-10 protocol design. At that time, PPSV23 was the most widely used adult pneumococcal vaccine in older adults, recognized for its established safety and effectiveness profile [26,27,61,62,63]. It has been commonly used (and was previously or is currently recommended) for the prevention of PD in older adults, as well as in certain younger adults with increased risk profiles, in several countries [27,61,64,65,66,67,68]. In addition, PPSV23 has been assessed as cost-effective for the vaccination or re-vaccination of older adults in several countries [69,70,71].

The current study adds to the body of knowledge regarding the impact of V116 in adults in regions with existing infant or childhood pneumococcal vaccination programs. STRIDE-10 complements findings obtained in STRIDE-6 that showed similar results in a vaccine-experienced population [29]. Consistent with the findings presented here comparing V116 against PPSV23, V116 demonstrated non-inferiority to common serotypes, superiority to nearly all unique serotypes, and a similar safety profile with respect to 20-valent PCV (PCV20) in STRIDE-3 [23]. Furthermore, in STRIDE-3, V116 demonstrated non-inferiority to common serotypes, superiority to nearly all unique serotypes, and a similar safety profile compared to PCV20 (PCV20 is a vaccine approved more recently than PPSV23, and is currently recommended in the United States for adults ≥50 years of age and for younger adults with underlying medical conditions or other risk factors) [23,27,72,73].

### Limitations

STRIDE-10 used an active-control design, rather than placebo as a control. Placebo-controlled clinical studies for new PCVs are no longer practical, and present an ethical challenge given the clinical efficacy and widespread use of licensed pneumococcal vaccines worldwide [17,74]. To allow for rigorous comparisons between the V116 and PPSV23 groups, this study excluded pneumococcal vaccine-experienced adults as well as adults who were immunocompromised; thus, the findings from this study cannot be generalized to these populations. However, V116 has been previously assessed in pneumococcal vaccine-experienced adults ≥50 years of age as part of the V116 clinical development program in STRIDE-6 [29].

Adults ≥75 years of age are at higher risk of PD than younger adults [6]. The proportion of adults in this age group in the current study was approximately 10%. This relatively low recruitment is partly because individuals in this age group are likely to have previously received a pneumococcal vaccine and would therefore have been excluded from the study. The goal of enrolling to ensure that more than half of participants were ≥65 years of age was met.

This study assessed the safety, tolerability, and immunogenicity of V116, but did not evaluate the effectiveness of the vaccine or its impact on PD. Nevertheless, functional immune responses measured by OPA are well-established surrogate measures of protection against PD, and, herein, V116 demonstrated acceptable such responses. The effectiveness of V116 in this population will need confirmation through future real-world observational studies. In addition, post-marketing experience will be important to further characterize the safety and effectiveness of V116 in larger, more representative real-world populations than those available in clinical trials [75,76].

## 5. Conclusions

The immunogenicity and safety profile of V116 in this study supports V116 as an adult-specific vaccine for the prevention of PD in adults. Residual PD in adults remains an unmet medical need. Based on the findings of this study, V116 may broaden disease coverage for PD in adults, when compared with PPSV23.

## Figures and Tables

**Table 1:** Baseline characteristics and demographics.

All Vaccinated Participants	V116 N = 739	PPSV23 N = 741
	n (%)	n (%)
**Sex**		
Female	407 (55.1)	410 (55.3)
Male	332 (44.9)	331 (44.7)
**Age (years)**		
Median (range)	65.0 (50–90)	65.0 (50–88)
50–64	342 (46.3)	342 (46.2)
65–74	326 (44.1)	328 (44.3)
≥75	71 (9.6)	71 (9.6)
**Race**		
White	453 (61.3)	467 (63.0)
Asian	151 (20.4)	140 (18.9)
Multiple	119 (16.1)	124 (16.7)
**Ethnicity**		
Hispanic or Latino	150 (20.3)	162 (21.9)
**Number of risk factors**		
No risk factor	538 (72.8)	523 (70.6)
One risk factor	169 (22.9)	184 (24.8)
≥2 risk factors	32 (4.3)	34 (4.6)

PPSV23: 23-valent pneumococcal polysaccharide vaccine; V116: 21-valent, adult-specific pneumococcal conjugate vaccine.

**Table 2:** Summary of AEs.

	V116 N = 739	PPSV23 N = 741
	n (%)	n (%)
**≥1 AE ^†^**	**451 (61.0)**	**421 (56.8)**
Mild (Grade 1)	304 (41.1)	285 (38.5)
Moderate (Grade 2)	119 (16.1)	111 (15.0)
Severe (Grade 3)	8 (1.1)	13 (1.8)
Potentially life-threatening (Grade 4)	20 (2.7)	12 (1.6)
Injection-site AEs	336 (45.5)	280 (37.8)
Systemic AEs	310 (41.9)	302 (40.8)
**≥1 solicited AE**	**369 (49.9)**	**359 (48.4)**
**Vaccine-related AEs ^‡^**	**395 (53.5)**	**354 (47.8)**
Injection-site AEs	335 (45.3)	280 (37.8)
Systemic AEs	196 (26.5)	185 (25.0)
**SAEs**	**22 (3.0)**	**18 (2.4)**
**Serious vaccine-related Aes ^‡^**	**0 (0.0)**	**0 (0.0)**
**Deaths**	**0 (0.0)**	**0 (0.0)**

^†^ Reported AEs include non-serious AEs that occurred within 30 days of vaccination and SAEs occurring from Day 1 through to completion of study participation. ^‡^ Determined by the investigator to be related to the vaccine. AE: adverse event; PPSV23: 23-valent pneumococcal polysaccharide vaccine; SAE: serious adverse event; V116: 21-valent, adult-specific pneumococcal conjugate vaccine.

## Data Availability

The data sharing policy, including restrictions, of Merck Sharp & Dohme LLC, a subsidiary of Merck & Co., Inc., Rahway, NJ, USA (MSD), is available at https://trialstransparency.msdclinicaltrials.com/policies-perspectives.aspx (accessed 13 February 2025). Requests for access to the clinical study data can be submitted via email to the Data Access mailbox (dataaccess@msd.com).

## Supplementary Materials

The following supporting information can be downloaded at: https://www.mdpi.com/article/10.3390/vaccines13040341/s1, Table S1. Participants with pre-specified medical history conditions associated with an increased risk of PD. Table S2. Proportions of participants in V116 with a ≥4-fold rise in OPA responses to cross-reactive serotypes 6C and 15B. Table S3. Solicited AEs by duration. Table S4. Summary of AEs stratified by age. Table S5. Summary of solicited AEs stratified by age. Figure S1. IgG GMCs for all 12 serotypes common to V116 and PPSV23 at 30 days post-vaccination. Figure S2. IgG GMCs for the nine serotypes unique to V116 at 30 days post-vaccination. Figure S3. OPA responses (GMFRs) for (A) 12 serotypes common to V116 and PPSV23 and (B) nine serotypes unique to V116, from Day 1 (pre-vaccination) to 30 days post-vaccination. Figure S4. OPA GMT ratios for all unique serotypes stratified by age.
