# Supplementary material for: A Phase 3 Randomized Trial Investigating the Safety, Tolerability, and Immunogenicity of V116, an Adult-Specific Pneumococcal Vaccine, Compared with PPSV23, in Adults ≥50 Years of Age (STRIDE-10)
Source: Vaccines (Basel). 2025 Mar 22;13(4):341. doi: 10.3390/vaccines13040341 (PMC12031205; doi:10.3390/vaccines13040341)
Supplement: Supplementary file 1 [file vaccines-13-00341-s001.zip › vaccines-3403617-supplementary.pdf]

Supplementary Information

Supplementary Table S1. Participants with pre-specified medical history conditions associated with an increased risk of PD

	V116		PPSV23		Total	
	n	%	n	%	n	%
**Participants in population**	739		741		1480	
**Participants by risk factors**						
**Alcoholism**	3	0.4	0	0.0	3	0.2
Alcoholism	3	0.4	0	0.0	3	0.2
**Chronic heart disease**	16	2.2	14	1.9	30	2.0
Cardiac failure congestive	12	1.6	11	1.5	23	1.6
Cardiomyopathy	9	1.2	8	1.1	17	1.1
**Chronic kidney disease**	10	1.4	12	1.6	22	1.5
Chronic kidney disease	9	1.2	9	1.2	18	1.2
Renal impairment	1	0.1	3	0.4	4	0.3
**Chronic liver disease**	13	1.8	21	2.8	34	2.3
Hepatic cirrhosis	0	0.0	1	0.1	1	0.1
Hepatic fibrosis	2	0.3	4	0.5	6	0.4
Hepatobiliary disease	11	1.5	17	2.3	28	1.9
**Chronic lung disease**	51	6.9	49	6.6	100	6.8
Asthma	41	5.5	35	4.7	76	5.1
Chronic obstructive pulmonary disease	11	1.5	12	1.6	23	1.6
Emphysema	1	0.1	3	0.4	4	0.3
**Diabetes**	87	11.8	89	12.0	176	11.9
Diabetes mellitus	87	11.8	89	12.0	176	11.9
**Smoking**	59	8.0	73	9.9	132	8.9
Smoking†	59	8.0	73	9.9	132	8.9
**Number of risk factors**						
Participants with no risk factor	538	72.8	523	70.6	1061	71.7

Participants with single risk factor	169	22.9	184	24.8	353	23.9
Participants with two or more risk factors	32	4.3	34	4.6	66	4.5

†Smoking only included current smokers.
The broad category of each medical history condition included one or more preferred terms based on MedDRA version 26.1. The number of risk factors was calculated based on the broad category.
MedDRA: Medical Dictionary for Regulatory Activities; PD: pneumococcal disease; PPSV23: 23-valent pneumococcal polysaccharide vaccine; V116: 21-valent, adult-specific pneumococcal conjugate vaccine.

Supplementary Table S2. Proportions of participants in V116 with a ≥4-fold rise in OPA responses to cross-reactive serotypes 6C and 15B

	V116 N = 739			
**Serotype**	**n**	**m**	**% (95% CI)†**	***p*-value†**
6C	620	327	52.7 (48.7, 56.7)	0.093
15B	588	427	72.6 (68.8, 76.2)	<0.001

Acceptability = lower bound of the 95% CI of the percentages of participants with a ≥4-fold rise in OPA responses from baseline to 30 days post-vaccination being >50 percentage points (1-sided *p*-value <0.025).
†Point estimate, 95% CI, and *p*-value are based on the Clopper–Pearson method.
CI: confidence interval; m: number of participants with the indicated response; N: number of participants randomized and vaccinated; n: number of participants contributing to the analysis; OPA: opsonophagocytic activity; PPSV23: 23-valent pneumococcal polysaccharide vaccine; V116: 21-valent, adult-specific pneumococcal conjugate vaccine.

Supplementary Table S3. Solicited AEs by duration

AE, n (%)	Duration	V116 (N = 739)	PPSV23 (N = 741)
**≥1 AE**	**≤3 days**	**308 (41.7)**	**297 (40.1)**
	>3 days and ≤5 days	53 (7.2)	52 (7.0)
	>5 days and ≤10 days	4 (0.5)	6 (0.8)
	>10 days	0 (0.0)	2 (0.3)
	Unknown	1 (0.1)	0 (0.0)
**Solicited injection-site AEs**			
Erythema	≤3 days	27 (3.7)	43 (5.8)
	>3 days and ≤5 days	8 (1.1)	3 (0.4)
	>5 days and ≤10 days	0 (0.0)	1 (0.1)
Pain	≤3 days	264 (35.7)	220 (29.7)
	>3 days and ≤5 days	23 (3.1)	37 (5.0)
	>5 days and ≤10 days	0 (0.0)	3 (0.4)
	>10 days	0 (0.0)	1 (0.1)
	Unknown	1 (0.1)	0 (0.0)
Swelling	≤3 days	32 (4.3)	29 (3.9)
	>3 days and ≤5 days	4 (0.5)	2 (0.3)
	>5 days and ≤10 days	0 (0.0)	1 (0.1)
**Solicited systemic AEs**			
Fatigue	≤3 days	98 (13.3)	102 (13.8)
	>3 days and ≤5 days	19 (2.6)	12 (1.6)
	>5 days and ≤10 days	4 (0.5)	2 (0.3)
	Unknown	1 (0.1)	0 (0.0)
Headache	≤3 days	87 (11.8)	80 (10.8)
	>3 days and ≤5 days	13 (1.8)	7 (0.9)
	>5 days and ≤10 days	0 (0.0)	1 (0.1)
	>10 days	0 (0.0)	1 (0.1)

	Unknown	1 (0.1)	0 (0.0)
Myalgia	≤3 days	37 (5.0)	41 (5.5)
	>3 days and ≤5 days	2 (0.3)	3 (0.4)

Every participant was counted a single time for each applicable specific AE and was classified according to the longest duration. Ongoing AEs were categorized as having duration >10 days. Solicited AEs were solicited from Day 1 to Day 5 post-vaccination.
AE: adverse event; PPSV23: 23-valent pneumococcal polysaccharide vaccine; V116: 21-valent, adult-specific pneumococcal conjugate vaccine.

Supplementary Table S4. Summary of AEs stratified by age

	50–64 years				65–74 years				≥75 years			
	V116		PPSV23		V116		PPSV23		V116		PPSV23	
	n	%	n	%	n	%	n	%	n	%	n	%
Participants in population	342		342		326		328		71		71	
With one or more AEs	232	67.8	217	63.5	179	54.9	171	52.1	40	56.3	33	46.5
Injection site	186	54.4	153	44.7	127	39.0	110	33.5	23	32.4	17	23.9
Systemic	157	45.9	154	45.0	127	39.0	123	37.5	26	36.6	25	35.2
With no AE	110	32.2	125	36.5	147	45.1	157	47.9	31	43.7	38	53.5
With vaccine-related[†] AEs	212	62.0	185	54.1	153	46.9	146	44.5	30	42.3	23	32.4
Injection site	185	54.1	153	44.7	127	39.0	110	33.5	23	32.4	17	23.9
Systemic	115	33.6	101	29.5	68	20.9	72	22.0	13	18.3	12	16.9
With serious AEs	5	1.5	8	2.3	14	4.3	9	2.7	3	4.2	1	1.4
With serious vaccine-related AEs	0	0.0	0	0.0	0	0.0	0	0.0	0	0.0	0	0.0
Death	0	0.0	0	0.0	0	0.0	0	0.0	0	0.0	0	0.0

[†]Determined by the investigator to have been related to the vaccine. All injection-site AEs and pyrexia (defined as maximum temperature ≥100.4 °F [38.0 °C] solicited from Day 1 through Day 5 post-vaccination) were considered vaccine-related.
Reported AEs included non-serious AEs within 30 days of vaccination and serious AEs occurring from Day 1 through 6 months post-vaccination.
AE: adverse event; PPSV23: 23-valent pneumococcal polysaccharide vaccine; V116: 21-valent, adult-specific pneumococcal conjugate vaccine.

Supplementary Table S5. Summary of solicited AEs stratified by age

	50–64 years				65–74 years				≥75 years			
	V116		PPSV23		V116		PPSV23		V116		PPSV23	
	n	%	n	%	n	%	n	%	n	%	n	%
**Participants in population**	342		342		326		328		71		71	
With one or more solicited AEs	210	61.4	188	55.0	134	41.1	145	44.2	25	35.2	26	36.6
With no solicited AEs	132	38.6	154	45.0	192	58.9	183	55.8	46	64.8	45	63.4
**Solicited injection-site AE**	180	52.6	153	44.7	108	33.1	110	33.5	18	25.4	17	23.9
Injection-site erythema	16	4.7	24	7.0	15	4.6	19	5.8	4	5.6	4	5.6
Injection-site pain	175	51.2	144	42.1	98	30.1	103	31.4	15	21.1	14	19.7
Injection-site swelling	18	5.3	18	5.3	16	4.9	13	4.0	2	2.8	1	1.4
**Solicited systemic AEs**	114	33.3	95	27.8	63	19.3	73	22.3	13	18.3	16	22.5
Fatigue	76	22.2	62	18.1	38	11.7	43	13.1	8	11.3	11	15.5
Headache	62	18.1	51	14.9	35	10.7	33	10.1	4	5.6	5	7.0
Myalgia	27	7.9	20	5.8	9	2.8	21	6.4	3	4.2	3	4.2
Pyrexia	4	1.2	4	1.2	3	0.9	4	1.2	1	1.4	2	2.8

Every participant was counted a single time for each applicable row and column. Injection-site erythema, injection-site pain, injection-site swelling, fatigue, headache, and myalgia were solicited from Day 1 through Day 5 post-vaccination. Pyrexia was defined as maximum temperature ≥100.4 °F (38.0 °C) solicited from Day 1 through Day 5 post-vaccination. MedDRA version 26.1 was used in the reporting of this study.
AE: adverse event; MedDRA: Medical Dictionary for Regulatory Activities; PPSV23: 23-valent pneumococcal polysaccharide vaccine; V116: 21-valent, adult-specific pneumococcal conjugate vaccine.

Supplementary Figure S1. IgG GMCs for all 12 serotypes common to V116 and PPSV23 at 30 days post-vaccination

Pneumococcal serotype	V116		PPSV23		GMC ratio
	n	GMC	n	GMC	(95% CI)
3	731	0.79	734	0.69	1.14 (1.04, 1.25)
7F	731	9.35	734	5.02	1.86 (1.65, 2.10)
8	731	13.83	734	11.85	1.17 (1.04, 1.31)
9N	731	11.21	734	7.23	1.55 (1.37, 1.76)
10A	731	15.05	734	7.41	2.03 (1.79, 2.31)
11A	731	8.83	734	4.01	2.20 (1.97, 2.46)
12F	731	2.28	734	1.14	1.99 (1.72, 2.30)
17F	731	16.39	734	6.85	2.39 (2.12, 2.70)
19A	731	9.54	734	6.42	1.49 (1.32, 1.67)
20A	731	22.97	734	12.32	1.86 (1.65, 2.10)
22F	731	5.32	734	3.00	1.77 (1.55, 2.02)
33F	731	19.12	734	15.70	1.22 (1.08, 1.37)

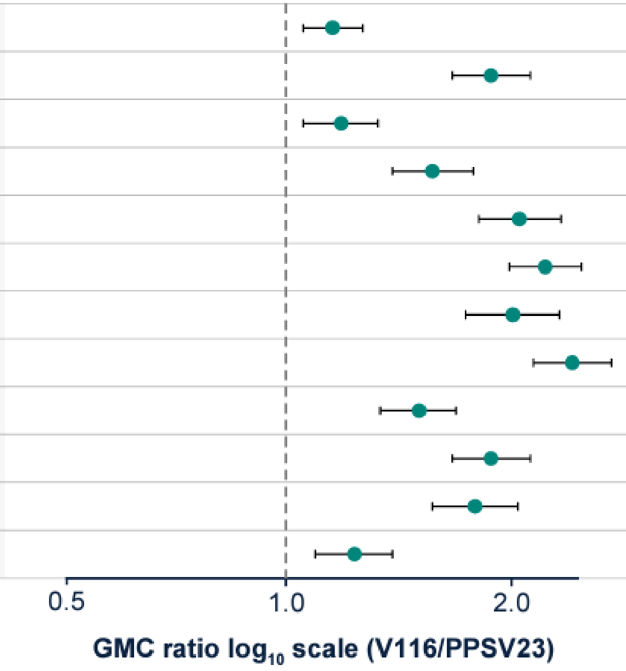

CI: confidence interval; GMC: geometric mean concentration (μg/mL); IgG: immunoglobulin G; PPSV23: 23-valent pneumococcal polysaccharide vaccine; V116: 21-valent, adult-specific pneumococcal conjugate vaccine.

Supplementary Figure S2. IgG GMCs for the nine serotypes unique to V116 at 30 days post-vaccination

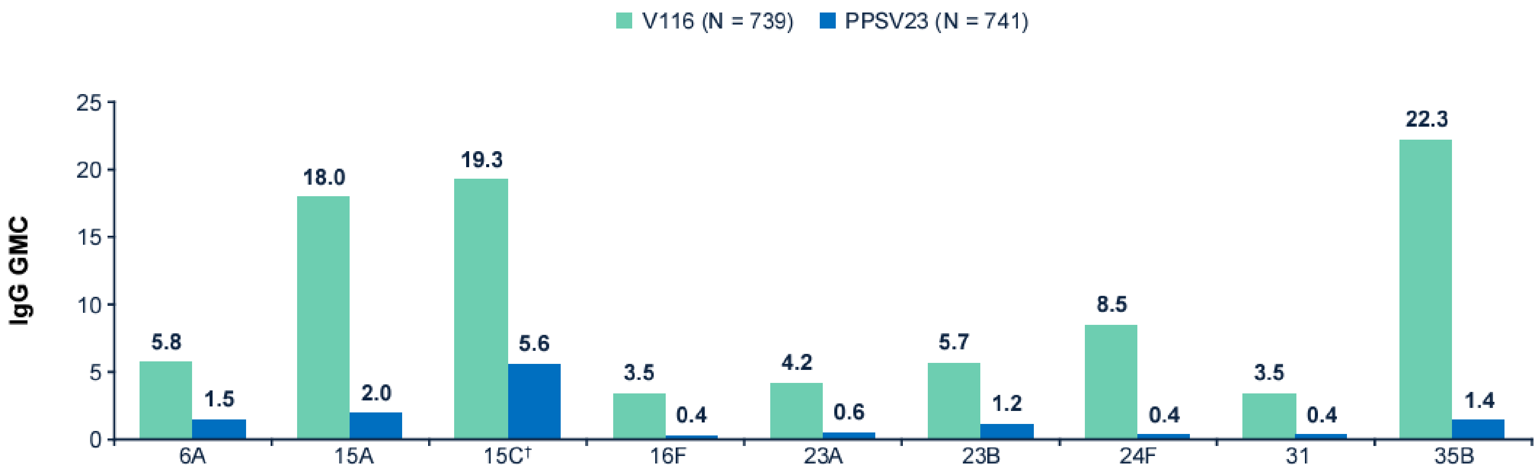

GMC ratio (V116/PPSV23) 95% CI	3.78 (3.29, 4.35)	8.92 (7.89, 10.09)	3.43 (3.01, 3.92)	9.84 (8.83, 10.97)	7.59 (6.68, 8.61)	4.79 (4.26, 5.38)	21.19 (18.98, 23.65)	8.69 (7.82, 9.65)	15.53 (14.13, 17.07)

GMCs, GMC ratio, and 95% CI are estimated from a cLDA model.

†Serotype 15C represented the immune response to the deOAc15B polysaccharide, as the molecular structure for deOAc15B and 15C were similar; anti-15C immune responses were assessed in this study.

CI: confidence interval; cLDA: constrained longitudinal data analysis; GMC: geometric mean concentration (µg/mL); IgG: immunoglobulin G; PPSV23: 23-valent pneumococcal polysaccharide vaccine; V116: 21-valent, adult-specific pneumococcal conjugate vaccine.

Supplementary Figure S3. OPA responses (GMFRs) for A) 12 serotypes common to V116 and PPSV23 and B) nine serotypes unique to V116, from Day 1 (pre-vaccination) to 30 days post-vaccination

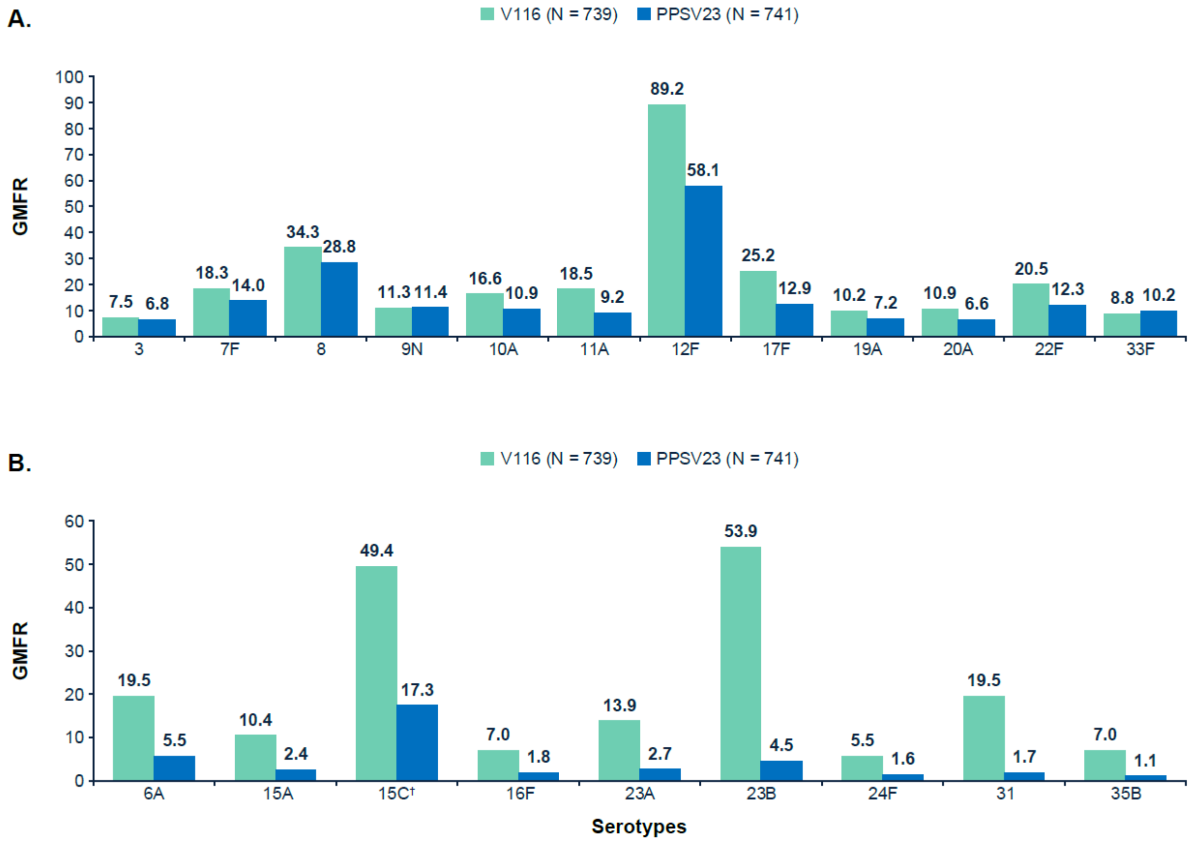

†Serotype 15C represented the immune response to the deOAc15B polysaccharide, as the molecular structure for deOAc15B and 15C are similar; anti-15C immune responses were assessed in this study.
GMFR: geometric mean fold rise; OPA: opsonophagocytic activity; PPSV23: 23-valent pneumococcal polysaccharide vaccine; V116: 21-valent, adult-specific pneumococcal conjugate vaccine.

Supplementary Figure S4. OPA GMT ratios for all unique serotypes stratified by age

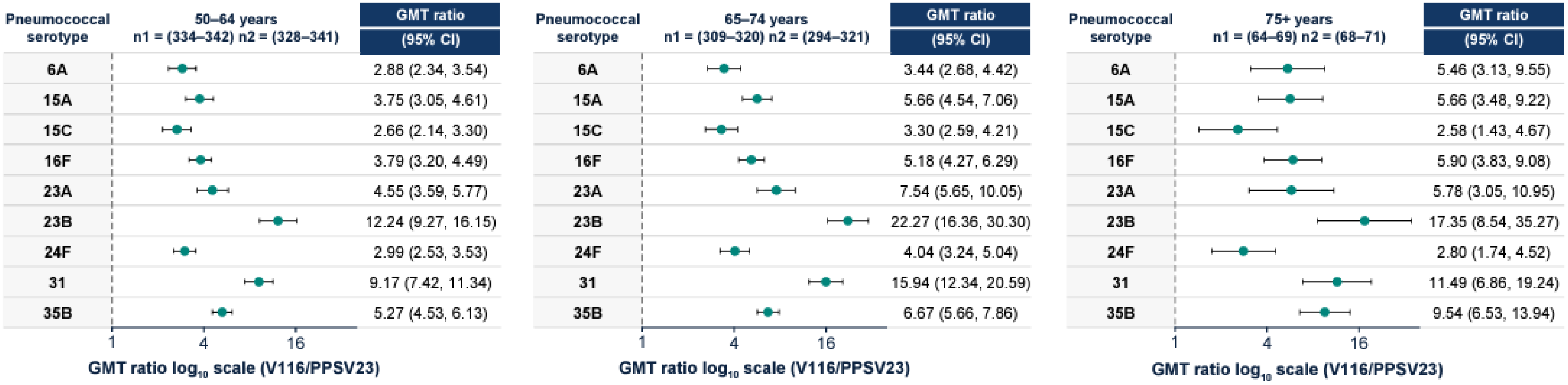

CI: confidence interval; GMT: geometric mean titer (1/dil); n1: number of participants contributing to the analysis from the V116 group across all unique serotypes; n2: number of participants contributing to the analysis from the PPSV23 group across all unique serotypes; OPA: opsonophagocytic activity; PPSV23: 23-valent pneumococcal polysaccharide vaccine; V116: 21-valent, adult-specific pneumococcal conjugate vaccine.
